# Supplementary material for: Tunable Human Myocardium Derived Decellularized Extracellular Matrix for 3D Bioprinting and Cardiac Tissue Engineering
Source: Gels. 2021 Jun 11;7(2):70. doi: 10.3390/gels7020070 (PMC8293197; doi:10.3390/gels7020070)
Supplement: Supplementary file 1 [file gels-07-00070-s001.zip › Supplementary/Supplementary Materials.pdf]

Supplementary Materials for

Tunable human myocardium derived decellularized extracellular matrix for 3D bioprinting and cardiac tissue engineering

Gozde Basara, S. Gulberk Ozcebe , Bradley W. Ellis and Pinar Zorlutuna*

*Correspondence: Pinar.Zorlutuna.1@nd.edu.

This PDF includes:
Materials and Methods
Figures S1 to S4

Other supplementary materials for this manuscript includes:
Movies S1 & S2

Materials and Methods:

Hydrogel Preparation with Visible light Crosslinker

Ruthenium (Ru) and Sodium Persulfate (SPS) solutions were prepared following the manufacturer's instructions (Advanced BioMatrix, Carlsbad, CA, USA). Simply, Ru was dissolved in 1X PBS at a concentration of 37.4 mg/mL and SPS was dissolved in 1X PBS at a concentration of 119 mg/mL. GelMA solution was prepared by dissolving GelMA (10% *w/v*) in PBS and Ru (2% *w/v*) and SPS (2% *w/v*) was included. Similarly, GelMA-MeHA solution was prepared by dissolving GelMA (20% *w/v*) and MeHA (2% *w/v*) in PBS and by mixing them in 1:1 ratio. Then Ru (2% *w/v*) and SPS (2% *w/v*) was included in the hydrogel mixture. 5 µL hydrogel solution was then placed on a stage in-between 100 µm thick spacers, and a glass slide was placed on top of the solution to achieve the required thickness. The hydrogel solution was then exposed to blue light for 3 s. Half of the gels were treated with mTGase solution for 30 mins at 37 °C, then the mTGase solution was replaced with PBS. The gels were kept at 37 °C overnight to achieve equilibrium swelling of the hydrogels.

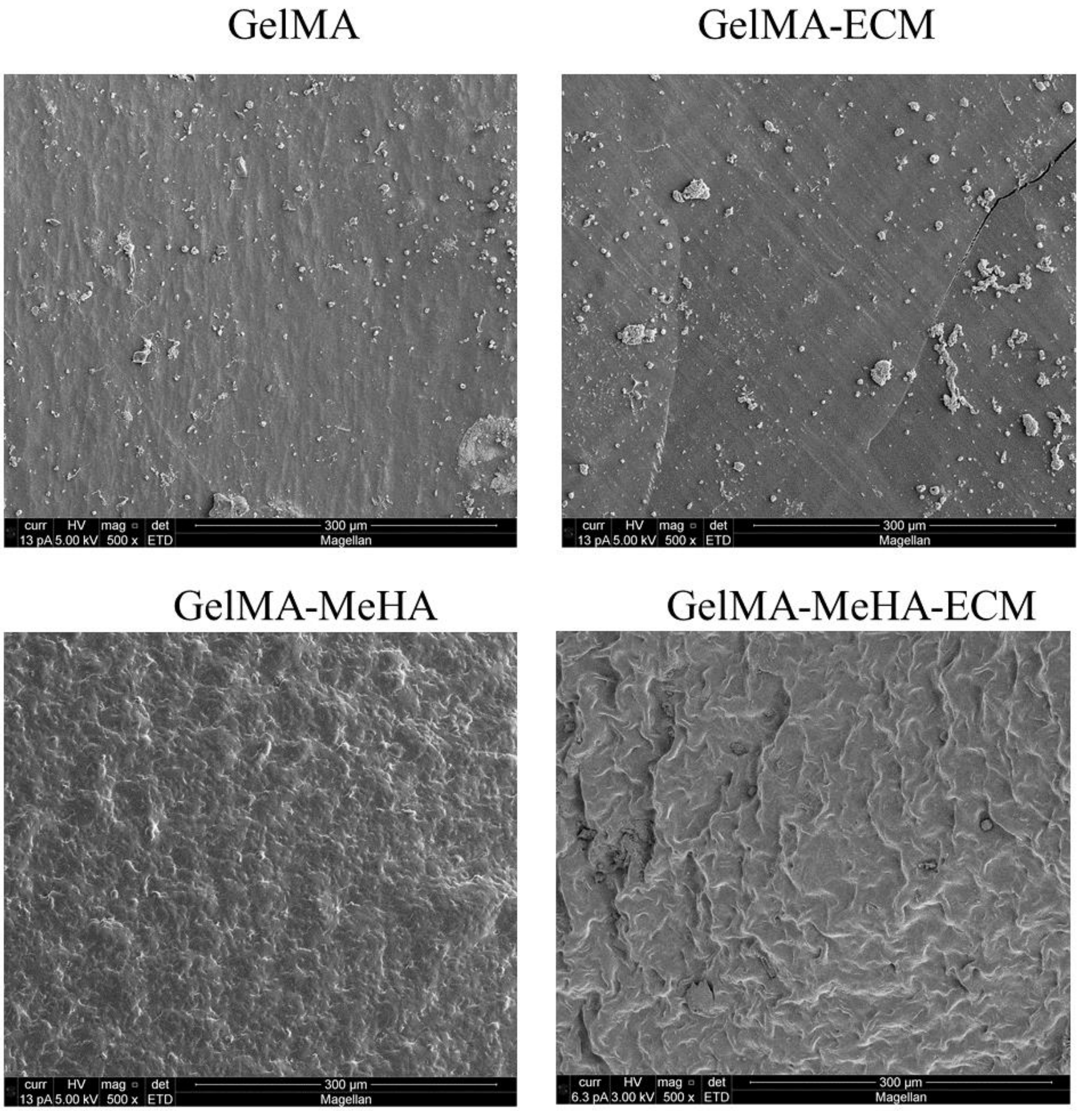

Figure S1. SEM images of GelMA, GelMA-dhECM, GelMA-MeHA, GelMA-MeHA-dhECM hydrogels.

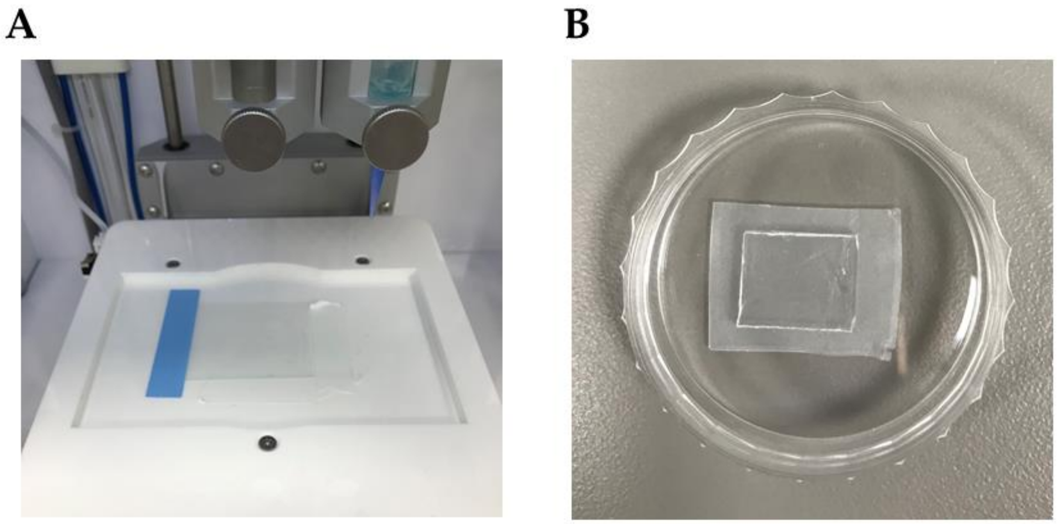

Figure S2. Parafilm coating (A) on the printing stage, (B) in the dish under the glass

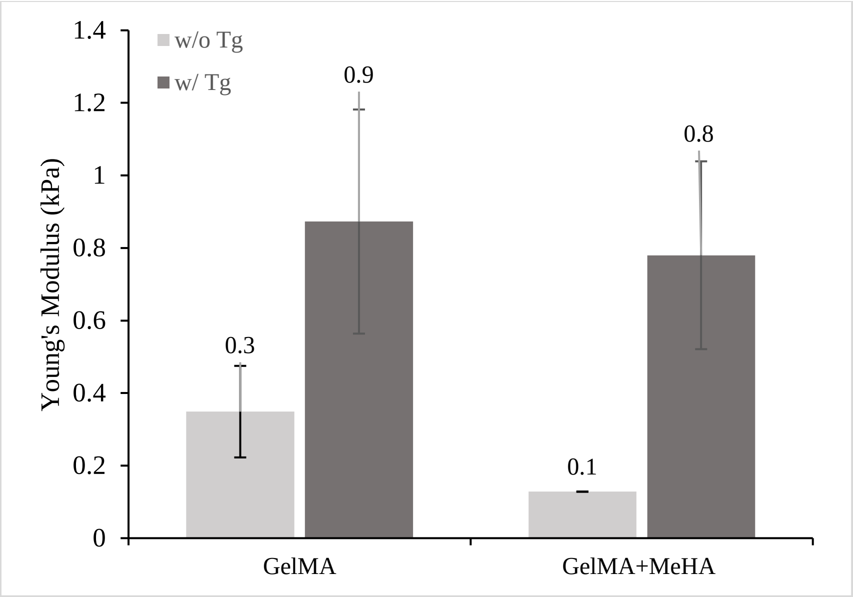

Figure S3. Young's Modulus of GelMA and GelMA-MeHA hydrogels with visible light crosslinking with and without mTGase treatment.

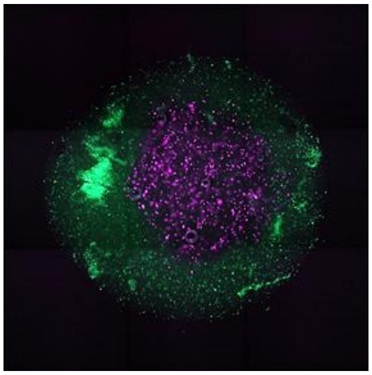

Figure S4. Printed boundary region using iCMs in GelMA-ECM bioink (green), and hCFs encapsulated in GelMA-MeHA-ECM bioink (magenta)
